# Membrane-associated GRP78 helps subgroup J avian leucosis virus enter cells

**DOI:** 10.1186/s13567-016-0373-6

**Published:** 2016-09-06

**Authors:** Lin Wang, Mei Mei, Aijian Qin, Jianqiang Ye, Kun Qian, Hongxia Shao

**Affiliations:** 1 Ministry of Education Key Lab for Avian Preventive Medicine, Yangzhou University, No. 12 East Wenhui Road, Yangzhou, 225009 Jiangsu China; 2 Key Laboratory of Jiangsu Preventive Veterinary Medicine, Yangzhou University, No. 12 East Wenhui Road, Yangzhou, 225009 China; 3 Jiangsu Co-innovation Center for Prevention and Control of Important Animal Infectious Diseases and Zoonoses, Yangzhou University, No. 12 East Wenhui Road, Yangzhou, 225009 China; 4 Jiangsu Key Lab of Zoonosis, Yangzhou University, No. 12 East Wenhui Road, Yangzhou, 225009 China; 5 National Research Center of Engineering and Technology for Veterinary Biologicals, Jiangsu Academy of Agricultural Sciences, Nanjing, 210014 Jiangsu China

## Abstract

We previously identified chicken Annexin A2 (chANXA2) as a novel receptor for retrovirus avian leucosis virus subgroup J (ALV-J), using a DF1 cell line expressing the viral envelope (env) protein. To further probe whether other proteins participate in virus infection, we investigated several host proteins from co-immunoprecipitation with the DF1 cell line expressing viral env. Mass spectrometry analysis indicates that the chicken glucose-regulation protein 78 (chGRP78) of the DF1 membrane interacted with the ALV-J env protein. The results revealed that antibodies or siRNA to chGRP78 significantly inhibited ALV-J infection and replication, and over-expression of chGRP78 enabled the entry of ALV-J into non-susceptible cells. Taken together, these results are the first to report that chGRP78 functions to help ALV-J enter cells.

## Introduction

Virus infection relies on the interaction between surface proteins and host receptors. In addition to virus receptors, other proteins may also interact with the virus and mediate infection. For example, HIV, a member of the retrovirus family, uses its glycoprotein gp120 to bind with CD4, CCR5 and CXCR4 as receptors to infect host cells [1–3]. Moreover, certain other proteins, such as CCR1, CCR8, CXCR6 (BONZO), GPR15 (BOB), GPR1, APJ, CX3CR1 (V28), CXCR5, and RDC1, also interact with gp120 and mediate virus entry into host cells [4–10]. Some of them were identified as co-receptors, which directly interact with virus and facilitate virus invasion to susceptible cells with greater efficiency.

ALV is another type of retrovirus. According to the antigenicity of its envelope glycoprotein, ALV can be divided into six subgroups (A, B, C, D, E, and J). The high variability of ALV-J’s envelope (env) protein not only distinguishes it from other subgroups, but also results in alternations of the virus’s pathogenicity, tumourigenicity and host range [11]. The env protein’s surface unit (SU) is a virus receptor binding-determining region. Currently, four cellular receptors for ALV have been identified. Tva [12], tvc [13], and chNHE1 [14] interact with the env proteins of ALV-A, C, and J, respectively. Recent research has identified chANXA2 as another receptor that is specific to ALV-J [15]. To further probe whether there are other proteins that specifically interact with ALV-J’s env protein to mediate viral infection, we used DF1 cell lines that over express ALV-J’s env protein to capture proteins of interest. Using immunological and molecular biological approaches, we identified 78-kDa glucose-regulated protein (GRP78) as a novel host protein that interacts with the env of ALV-J and is involved in the infection of DF1 cells by ALV-J. GRP78 is also referred to as Bip/HSPA5. This protein consists of membrane-associated [16, 17] and trans-membrane segments [18]. More than a ER stress-regulating chaperone [19], GRP78 participates in several biological or immunological processes and extensively influences virus infection or pathogenicity [20–23]. Meanwhile, GRP78 has been identified as a receptor for different types of virus, such as coxsackie B [24] and dengue fever virus [25], which directly interact with viral protein. Our results firmly establish that GRP78 can interact with the env of ALV-J to aid the entry of ALV-J into cells.

## Materials and methods

### Cells and virus

DF1 cells and pcDNA-env_DF1 cells [26] were maintained in Dulbecco’s modified Eagle’s medium (DMEM) supplemented with 5% fetal bovine serum and 1% antibiotics. HEK293T cells and GEF (Goose embryo fibroblast) cells were maintained in DMEM supplemented with 10% fetal bovine serum and 1% antibiotics. The ALV-J strain (JS09GY07) and ALV-A strain (AH10) were preserved in our laboratory.

### Antibodies and primers

Monoclonal antibody JE9, which specifically recognizes ALV-J’s gp85, was employed in the indirect immunofluorescence (IFA), Western blot and immunoprecipitation experiments to detect or capture ALV-J’s env protein [27]. Monoclonal antibody 5D3, which specifically recognizes ALV’s group specific protein p27, was employed in Western blot. A polyclonal antibody for chGRP78 was purchased from Santa Cruz Biotechnology, the product code is sc-1051 (Dallas, USA). Monoclonal antibody chicken β-actin (Santa Cruz Biotechnology, Dallas, USA) was used as an internal control in the Western blot. FITC-labelled goat anti-mouse antibody (Sigma, St. Louis, MO, USA) and HRP-labelled goat anti-mouse antibody (Sigma) were used as secondary antibodies. The primers used in the real-time PCR and the siRNA used to inhibit the expression of chGRP78 are listed in Table 1. The siRNA was synthesized by Invitrogen (Carlsbad, California, USA).

**Table 1:** **Sequences of the primers used for real-time PCR and the siRNA against chGRP78**

	Name	Sequence(5′–3′)
RT-PCR	ALV-J gp37	Forward: TGCGTGCGTGGTATTATTTC
		Reverse: AATGGTGAGGTCGCTGACTGT
	chGRP78	Forward: GACGATGAGGAGAAAAAGGAG
		Reverse: TGAATACACCCACACAAGAAT
	Chicken 18s	Forward: TCAGATACCGTCGTAGTTCC
		Reverse: TTCCGTCAATTCCTTTAAGTT
	Human β-actin	Forward: CACGAAACTACCTTCAACTCC
		Reverse: CATACTCCTGCTTGCTGATC
siRNA	366	Forward: AGGACAUCAAGUAUCUGCCCUUCAA
		Reverse: UUGAAGGGCAGAUACUUGAUGUCCU
	611	Forward: GGGUUGAACGUGAUGCGCAUUAUUA
		Reverse: UAAUAAUGCGCAUCACGUUCAACCC
	519	Forward: CCCACAGAUUGAAGUUACCUUUGAA
		Reverse: UUCAAAGGUAACUUCAAUCUGUGGG

### Co-immunoprecipitation and mass spectrometry

pcDNA-env_DF1 cells in 100 mm dishes were harvested by scraping with a rubber policeman and homogenised with NP-40 lysis buffer containing 25 mM Tris, 150 mM NaCl, 1 mM EDTA, 1% NP-40, 5% glycerol (pH7.4) and a protease inhibitor cocktail (Roche, Basel, Switzerland) incubating for 20 min, then the membrane proteins in the supernatant were sedimented by an additional spin at 13 200 *g* for 1 h at 4 °C and resuspended in 1% NP-40 lysis buffer. The membrane proteins from the pcDNA-env_DF1 cells were immunoprecipitated with the monoclonal antibody JE9, which is specific to ALV-J Env and Resin A (Thermo Scientific, Massachusetts, USA). Precipitated proteins were separated by SDS-PAGE. The gel was stained with a Silver Stain Kit for MS (Thermo Scientific). The bands of interests were collected and analysed using mass spectrometry [15].

### Antibody blocking assay

The DF1 cells were pre-treated with anti-GRP78 antibody which was diluted with DMEM at a concentration of 5, 25 and 50 μg/mL at 37 °C for 2 h and subsequently challenged with ALV-J at a MOI of 5 and incubated at 37 °C for 2 h. The DF1 cells were pre-treated with anti-GRP78 antibody which was diluted with DMEM at a concentration of 50 μg/mL and subsequently challenged with ALV-A at a MOI of 5 and incubated at 37 °C for 2 h. The cells were treated with acid glycine (pH 3.0) for 1 min to inactivate the non-internalized virus. After a wash with PBS, the cells were maintained in DMEM with 1% foetal calf serum and antibody at the concentrations mentioned above for 48 h. The replication of ALV-J in the treated cells was analysed with IFA, real-time PCR, Tissue culture infective dose 50 (TCID_50_) and Western blot, and the replication of ALV-A in the treated cells was analysed with Western blot.

### RNA interference assay

DF1 cells were transfected with stealth siRNA (50 pmol) against chGRP78. Six hours later, the cells were infected with ALV-J at a MOI of 1 and incubated at 37 °C for 2 h. Then, the cells were maintained in DMEM with 1% fetal bovine serum for 72 h. The interference effects of the siRNA directed against chGRP78 were evaluated with real-time PCR. Simultaneously, the replication of ALV-J in the DF1 cells was analysed with Western blot and TCID_50_ titration of the ALV-J in the collected cell culture supernatant.

### Infection of chGRP78-transfected cells with different concentration

First, we inserted the 1982 bp full-length chGRP78 coding sequence into pcDNA3.1; human 293T cells were transfected with different concentrations of pcDNA3.1-chGRP78 (0.5, 1.5, 3.0 and 4.5 μg respectively) and also transfected with 4.5 μg pcDNA3.1 as the control. Forty-eight hours later, the transfected cells were infected with ALV-J at a MOI of ten for 2.5 h. Subsequently the cells were treated with acid glycine (pH 3.0) for 1 min and washed three times with PBS. Then, the 293T cells were maintained in DMEM with 1% foetal bovine serum for 48 h. The cells were extracted RNA and elevated with real-time PCR.

### Infection of chGRP78-transfected cells

GEF cells were transfected with pcDNA3.1-chGRP78. The cells were transfected with pcDNA3.1 as the control. Forty-eight hours later, the transfected cells were infected with ALV-J at a MOI of ten for 2.5 h. Then, the GEF cells were maintained in DMEM with 1% foetal bovine serum. After 6 days later, the replication of ALV-J was analysed with IFA using JE9 antibody.

### Real-time PCR

RNA was isolated from whole-cell lysates using a Total RNA Miniprep Kit (Axygen, California, USA). The RNA was reverse transcribed using a PrimerScript RT Reagent Kit (Takara, Kusatsu, Japan) with primers targeting the ALV-J gp37 and chGRP78 coding sequences. Chicken 18s and human β-actin were used as an internal control in the real-time PCR. Fold change of target gene RNA level was calculated with the 2^−△△Ct^ method. GraphPad Prism 5.0 (GraphPad Software) was used to analyse the data. Data is expressed in column style with mean ± SD and statistic variance was analysed using the One-way ANOVA test. *P* < 0.05 was considered as significant.

### TCID_50_ assay

To determine the TCID_50_ level of the ALV-J in the collected supernatants, DF1 cells were plated in 96-well plates (2.5 × 10^4^ cells/well). Twenty-four hours later, the supernatants from antibody blocking assays or RNA interference assays were continuously diluted from 10- to 10^10^-fold. Each dilution was used to infect the DF1 cells in eight duplicate wells. After incubation at 37 °C for 2.5 h, the supernatants were replaced with DMEM containing 1% foetal bovine serum. Seven days later, the cells were immobilized, and IFA was conducted with JE9. The final dilution of virus TCID_50_ was calculated according to the Reed-Muench formula.

### IFA

The treated DF1 cells and GEF cells were immobilized with 4% paraformaldehyde and then treated with 0.25% Triton X-100. After incubation with PBS containing 2% BSA for 45 min, the cells were incubated with JE9 as the primary antibody. Then, FITC-labelled goat anti-mouse antibody was used as the secondary antibody. Finally, Hoechst 33342 was used to stain the cell nuclei at room temperature for 2 min. The cells were stored in 50% glycerine, and the fluorescence signals were observed under confocal microscopy.

### Western blot of env expression in DF1 cells

The expression levels of ALV-J env protein were also detected with Western blot. Following infection with ALV-J for 48 h, the DF1 cells were harvested and lysed with 1% NP-40 and a protease inhibitor cocktail. The cell lysates were used for SDS-PAGE and then transferred to nitrocellulose membranes for Western blot analysis. JE9, 5D3 and β-actin were employed as the first antibody respectively, and HRP-labelled goat anti-mouse antibody was employed as the second antibody. The membrane was finally immersed in Super Signal West Pico (Thermo, Massachusetts, USA), and the chemiluminescent signals were observed with a FluorChemE imaging system (Protein Simple, California, USA).

## Results

### chGRP78 bound the env protein of ALV-J

**Figure 1:**
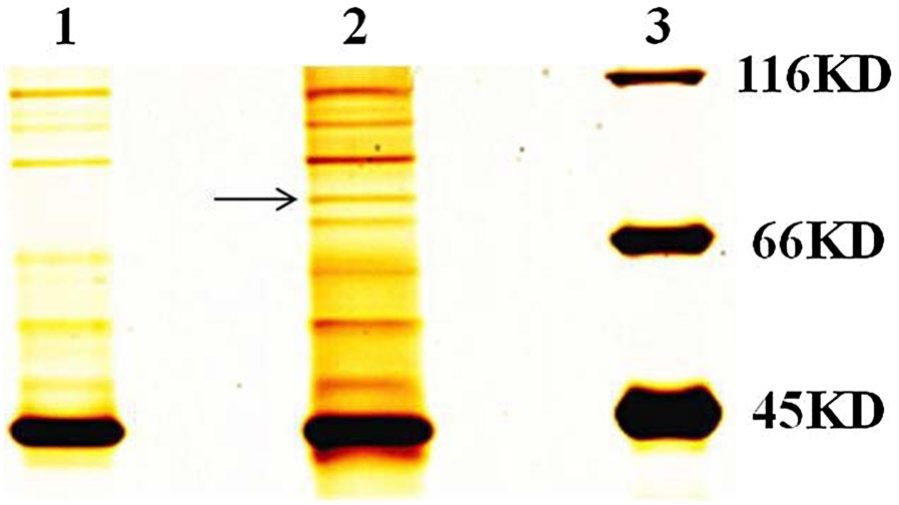
**Silver staining of the protein precipitates for the membrane proteins of the pcDNA-env_DF1 cells.** Lane 1, precipitated with isotype control IgG; lane 2, precipitated with JE9; lane 3, protein marker.

To identify a novel ALV-J-binding protein in the DF1 cells, the pcDNA-env_DF1 cell line expressing ALV-J env protein was used in co-immunoprecipitation assays (co-IP). The pcDNA-env_DF1 cells were lysed and then performed immunoprecipitation with the single monoclonal antibody (mAb) JE9, which is specific to ALV-J env. Silver staining for SDS-PAGE of the immunoprecipitation revealed several different bands in the lysate that was immunoprecipitated with ALV-J-specific mAb JE-9 and not with the control antibody (Figure 1). Mass spectrometry further revealed that one of these bands was chGRP78 which was 78-kDa.

### The antibody against chGRP78 significantly inhibited ALV-J infection and replication

**Figure 2:**
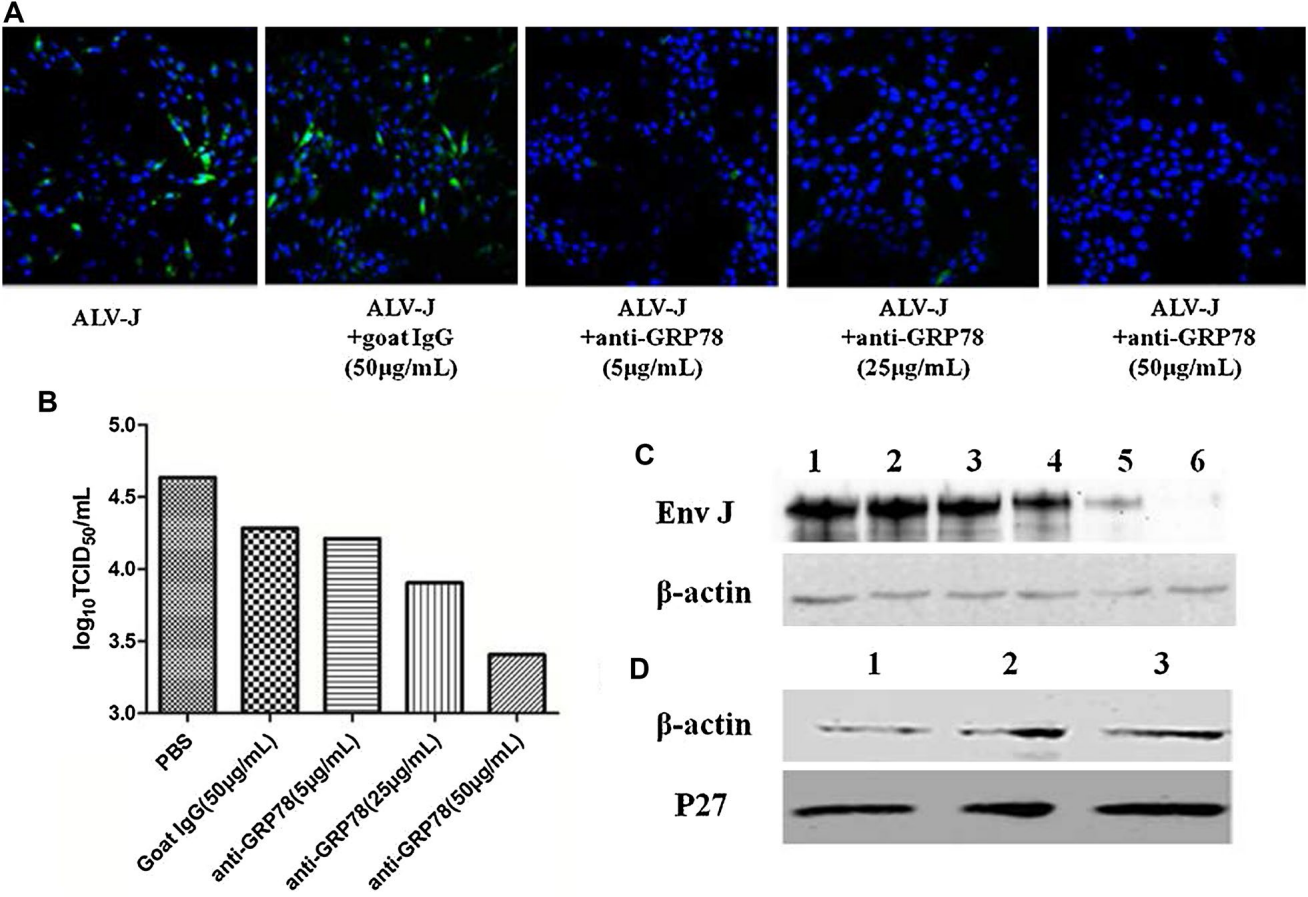
**Inhibition of ALV-J infection by antibodies to GRP78.** The DF1 cells that had been pre-treated with antibodies against chGRP78 were infected with ALV-J, and ALV-J replication in the treated cells was analysed. **A** IFA analysis using JE9; **B** TCID_50_ analysis for viral titres; **C** Western blot analysis for the expression of env from ALV-J in the DF1 cells that had been treated with antibodies. Lane 1, blank; lane 2, mock; lane 3, goat IgG (50 μg/mL); lane 4, anti-GRP78 (5 μg/mL); lane 5, anti-GRP78 (25 μg/mL); lane 6, anti-GRP78 (50 μg/mL); **D** Western blot analysis of the expression of ALV-A p27 in the DF1 cells treated with antibodies. Lane 1, anti-GRP78 (50 μg/mL); lane 2, goat IgG (50 μg/mL); lane 3, mock.

We used antibodies against chGRP78 to perform blocking assays to evaluate the effects of chGRP78 on ALV-J infection in DF1 cells. Our results revealed that the viral infection/replication of ALV-J was significantly inhibited in groups that had been treated with anti-GRP78. As shown in Figure 2A, there was little visible immunofluorescence in the cells that were treated with 50 or 25 μg/mL of the antibody against chGRP78 in the IFA. Moreover, only a few positive cells were found among the cells that were treated with 5 mg/mL of antibody against chGRP78. In contrast, many positive cells were found among the cells that were treated with the control IgG and among the untreated cells., the viral titres of the cells that were treated with 50 and 25 μg/mL of antibody against chGRP78 were approximately 50- and 10-fold less, respectively, than those of the cells that were treated with the control IgG being consistent with the IFA results (Figure 2B). The inhibitory effect on ALV-J infection/replication conferred by the antibody against chGRP78 was also confirmed by Western blot (Figure 2C). As a viral control, we also performed a blocking assay for ALV-A infection in the DF1 cells. As described in Figure 2D, the p27 expression levels of ALV-A in the groups that were treated with the antibody against chGRP78 were similar to those of the mock group, which indicates that the antibody against chGRP78 could not inhibit ALV-A infection/replication in DF1 cells. These data clearly demonstrate that blocking chGRP78 with a specific antibody can effectively and specifically inhibit the infection/replication of ALV-J.

### RNA interference with chGRP78 in DF1 cells inhibited ALV-J infection

**Figure 3:**
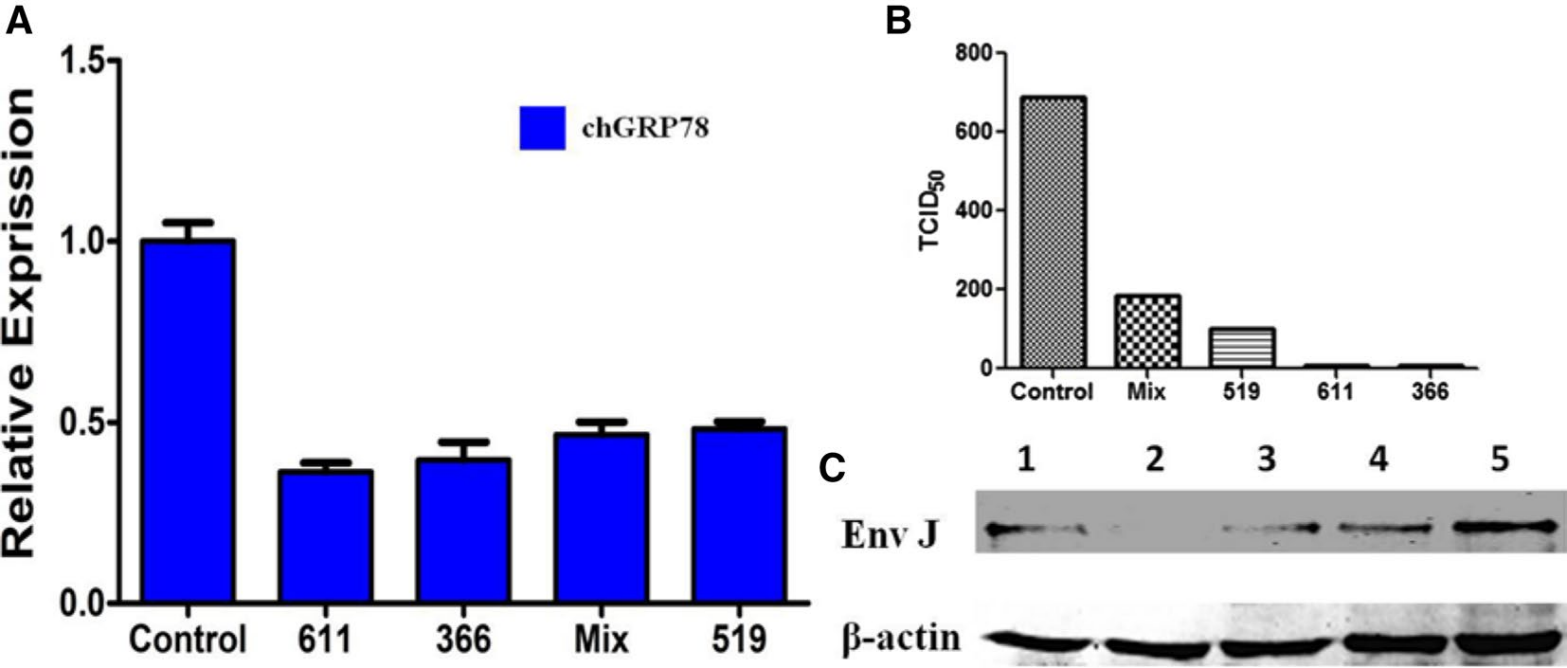
**siRNA against chGRP78 in DF1 cells.** The cells were transfected with siRNA (50 pmol) against chGRP78 for 6 h, and the cells were then infected with ALV-J at an MOI of 1 for 72 h. **A** The siRNA effects on chGRP78 were detected with real-time PCR. **B** The infection/replication of ALV-J was analysed with Western blot. Lanes 1, 2, 3 and 4, DF1 cells transfected with siRNA 519, 611, 366 and Mix (co-transfected with 519, 611 and 366), respectively, against chGRP78. Lane 5, DF1 cells transfected with control siRNA. **C** TCID_50_ analysis of the viral titres.

To further demonstrate that the chGRP78 protein plays an important role in ALV-J infection, an RNA interference assay was performed. Three pairs of siRNA were employed to reduce chGRP78 expression. Real-time PCR indicated that the mRNA level of chGRP78 was significantly inhibited in the DF1 cells (Figure 3A). Moreover, the Western blot results for the ALV-J envelope protein and the TCID_50_ titration of ALV-J in the DF1 cell supernatant revealed that virus replication was inhibited (Figures 3B and C). These results suggest that chGRP78 plays a significant role in the process of ALV-J infection of the host cell.

### chGRP78 over-expression allowed the ALV-J into non-permissible cells

**Figure 4:**
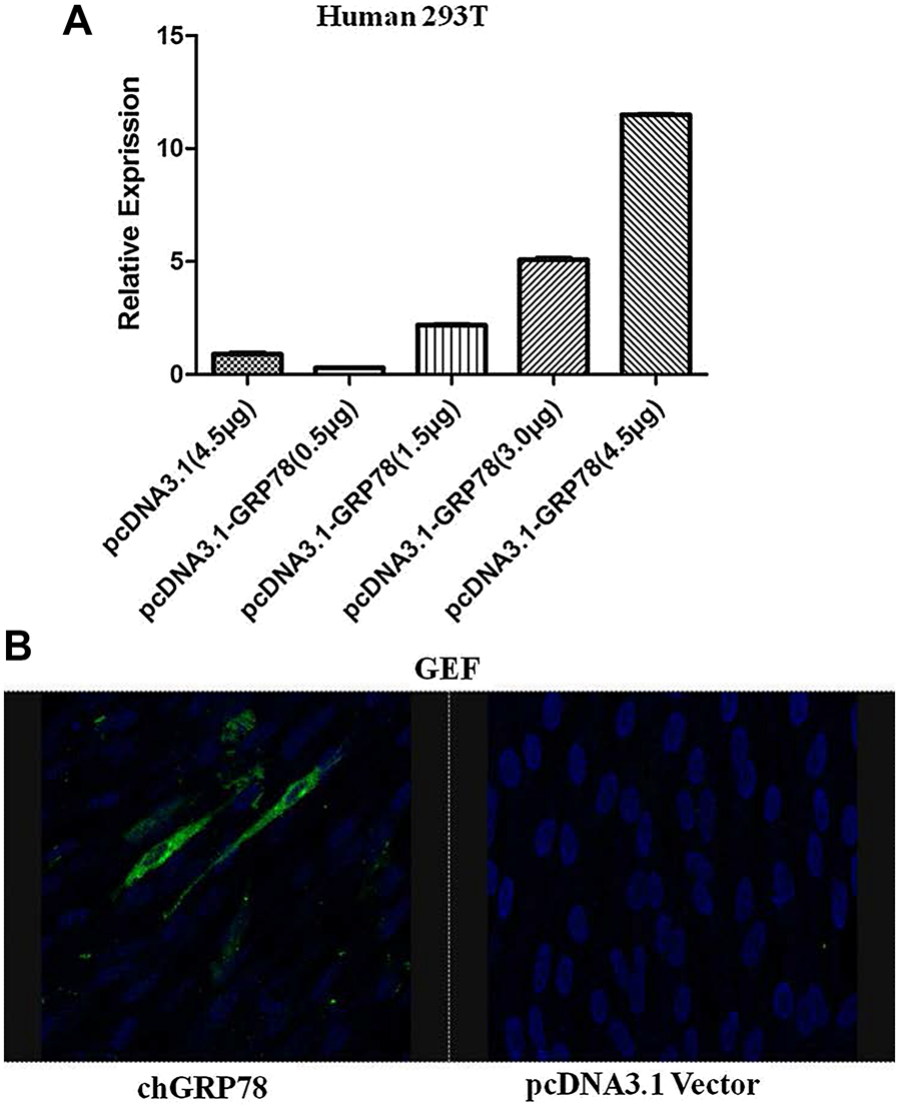
**Infection of the chGRP78-transfected cells.** 293T cells and GEF cells were transfected with chGRP78, and the transfected cells were then infected with ALV-J. **A** The replication of ALV-J in the 293T cells transfected with different concentrations of chGRP78 was measured by real-time PCR; **B** The replication of ALV-J in the GEF cells transfected with chGRP78 was measured by IFA.

Human 293T cells, which are non-susceptible to ALV-J, were transfected with different concentrations of pcDNA3.1-chGRP78 (0.5, 1.5, 3.0 and 4.5 μg) respectively, and transfected with 4.5 μg pcDNA3.1 as the control. After 48 h, the transfected cells were infected with ALV-J at an MOI of 5. The viral gp37 gene was detected by real-time PCR. We found that the mRNA level of ALV-J gp37 increased apparently with the concentration increasing, a significant positive correlation (Figure 4A). To further confirm this finding, we also transfected chGRP78 into GEF cells in which the ALV-J could not grow [28]. After 48 h, the transfected GEF were infected with ALV-J. The GEF cells were maintained in DMEM medium containing 1% foetal bovine serum for 6 days. Interestingly, as the IFA shows in Figure 4B, virus was recovered from the GEF cells transfected with chGRP78, but no virus was obtained from the GEF cells that were transfected with the control plasmid. Then, the infected GEF and supernatant were collected to recover ALV-J on DF1 cells. As excepted, the virus could be detected on DF1 cells by IFA. Together, these data demonstrate that the over-expression of chGRP78 in 293T or GEF cells can support ALV-J entry into these ALV-J non-permissible cells.

## Discussion

Virus attachment and entrance into host cells rely on host receptors. Occasionally, other host proteins may assist in the virus infection. For example, host glycosaminoglycans have been implicated in the mediation of the attachment and entry of mosquito-borne flaviviruses into mammalian cells [29, 30]; however, the corresponding function has not been elucidated. Previous studies with Japanese encephalitis virus (JEV) have reported the possible involvements of a 57-kDa protein derived from BHK-21 cells, the GAG protein from CHO cells and a 74-kDa molecule from Vero cells as possible receptors [31]. Subsequently, another report hypothesized that vimentin, an important cellular protein, is responsible for the attachment and entry of JEV into porcine cells [32].

Similarly, in addition to the identified host receptors chANXA2 and chNHE1, we found that chGRP78 is another important protein for AVL-J infection of DF1 cells. chGRP78 is a member of the HSP70 family that is located in both the membrane and plasma of various types of cells [33]. Despite its immune and biological functions [34], substantial research has found that chGRP78 is a receptor for pathogens such as dengue virus [35], coxsackie virus [24] and herpes simplex virus [36]. The roles of chGRP78 in the infections of other pathogens have been elucidated [37–39]. Collectively, this research indicates that chGRP78 may also play a role in avian pathogen infection. In the present study, we found that ALV-J replication was significantly inhibited when the DF1 cells were blocked with polyclonal antibody against chGRP78 or the expression of chGRP78 was interfered with via siRNA. The inhibition efficiency in DF1 cells is the same as that in chANXA2 [15]. This finding indicates that chGRP78 may have a similar function as chANXA2 in AVL-J infection. The over-expression of chGRP78 could permit the entry of ALV-J into 293T cells. Our study shows that the RNA amount of gp37 was increased in the first 48 h in 293T cells which were transfected with chGRP78, while other conditions were the same. In addition, ALV-J infection efficiency was also dose-dependent on chGRP78 expression in 293T cells. The gain of ALV-J susceptibility in GEF further supports the conclusion that chGRP78 may be a novel functional co-receptor for ALV-J infection. This finding indicates the importance of chGRP78 in AVL-J entry into host cells. However, the potential interaction among chNHE1, chANXA2 and chGRP78 need to be investigated, and the mechanism by which chGRP78 introduced ALV-J into the host cells remains unclear. Both of these issues are the subject of our future research. This research will also provide additional information regarding retrovirus prevention and control.
